# Emotional and Literacy Adaptation in Large Language Model–Based Medical Guidance: Development and Usability Study

**DOI:** 10.2196/100369

**Published:** 2026-10-28

**Authors:** Fran Casino, Antoni Martínez-Ballesté, Edgar Batista

**Affiliations:** 1 Department of Computer Engineering and Mathematics Universitat Rovira i Virgili Tarragona, Catalonia Spain

**Keywords:** conversational agents, large language models, adaptive communication, emotional sensitivity, literacy, human-machine interaction, context awareness

## Abstract

**Background:**

Large language models (LLMs) are increasingly deployed as conversational agents for communication and guidance, but their ability to adapt responses to a patient’s emotional state and literacy level remains insufficiently characterized. In patient-facing scenarios, effective communication requires not only accurate medical content but also appropriate tone, clarity, and responsiveness to user vulnerability.

**Objective:**

This study aimed to evaluate, within a controlled, single-scenario experimental design, the ability of contemporary LLMs to adapt medical guidance responses to different levels of emotional sensitivity and literacy, and to assess their consistency in both generating and interpreting such adaptations.

**Methods:**

A controlled benchmark was designed to evaluate how 4 LLMs adapt medical guidance responses to predefined emotional and literacy levels for a realistic patient profile asking an open-ended question. To establish a robust performance baseline, one model was deployed locally (Gemma 3 27B), and 3 are cloud-based (Claude Opus 4.5, Gemini 3 Pro, and GPT 5.2). Each model was tested across all combinations of 5 emotional levels, 3 literacy levels, and 3 prompt-verbosity variants, yielding 45 responses per model. Generated responses were assessed against human-reviewed ground truth for emotional and literacy alignment. A separate self-evaluation procedure measured each model’s ability to interpret its own outputs using the same adaptation framework. The study also examined generation-interpretation consistency and quantitative text properties, with particular attention to response length and verbosity.

**Results:**

Literacy adaptation was consistently accurate across models, often achieving near-zero error, indicating strong capability in adjusting explanatory complexity. In contrast, emotional adaptation showed greater variability and was more sensitive to model architecture and prompt design. The larger cloud-based models demonstrated more stable performance overall, while the local model exhibited higher variability, particularly under shorter prompts. A systematic asymmetry was observed between generation and interpretation. The models frequently misclassified low emotional and literacy levels as “normal,” suggesting limitations in recognizing user needs. Quantitative analysis revealed that response length increased with both emotional sensitivity and literacy level, with prompt verbosity and model-specific tendencies acting as secondary factors.

**Conclusions:**

Within our scenario, current LLMs demonstrated the capacity to support adaptive medical guidance, but their performance was uneven across dimensions, especially in terms of emotional generation and interpretation. For health care applications, these systems should be treated as assistive tools under human oversight rather than as autonomous communicators, with careful consideration of ethical, privacy, and safety implications in sensitive health contexts.

## Introduction

### Background

Conversational agents are increasingly integrated into digital health ecosystems, where they serve as intermediaries between complex clinical knowledge and patient understanding. The transition from rule-based systems to generative AI (GenAI) chatbots powered by large language models (LLMs) has significantly expanded their capabilities, enabling more flexible dialogue management, improved contextual reasoning, and more natural interactions [1]. As a result, conversational agents are being adopted across a broad range of patient-support applications, including mental health, chronic disease management, primary care follow-up, and older adult support, where they assist with psychoeducation, guided self-reflection, patient engagement, postvisit question answering, symptom clarification, and care-instruction support [2,3]. This growing use is reflected in recent adoption estimates. Around 22% of US adults report occasional use of AI chatbots for health care purposes, rising to 32% among those aged 18-29 years [4], while more than 5% of all ChatGPT messages globally are health care–related, with over 40 million users seeking health information each day [5].

Despite this rapid uptake, linguistic fluency alone is insufficient in patient-facing environments. Effective conversation requires interactions that are not only accurate and coherent but also understandable, context-sensitive, and aligned with patients’ varying needs [6]. In clinical practice, practitioners routinely adapt explanations through clarifications, simplified language, and an empathetic tone to ensure comprehension and trust. For instance, prior work suggests that women tend to prefer more empathetic interactions, whereas men often prefer more direct and factual communication [7]. LLM-based conversational agents must similarly emulate these adaptive behaviors to preserve safety and accessibility because misaligned communication can contribute to avoidable errors and reduce the effectiveness of patient support [8,9]. This perspective is consistent with emerging usability-by-design principles for health care LLMs, which emphasize that technology should adapt to patients rather than requiring patients to adapt to the technology [10].

A key challenge in this context is the persistent “one-size-fits-all” behavior of general-purpose LLMs, which lack explicit mechanisms to tailor communication to individual differences [7,11]. Although recent studies suggest that LLM-based conversational agents can perform comparably to human-delivered interventions [12,13], their nontechnical communication skills remain insufficiently characterized [14-18]. Two interrelated dimensions are particularly important. First, effective interaction requires sensitivity to the user’s emotional state, including appropriate modulation of tone, empathy, and affective framing. Second, effective interaction requires adapting the complexity of information delivery—including vocabulary, sentence structure, informational density, explanatory depth, and structural clarity—to the user’s literacy level and capacity to interpret and follow recommendations. Together, these dimensions constitute a central requirement for human-centered medical conversational agents: the ability to manage informational content and interpersonal sensitivity simultaneously.

Recent work has begun to explore these adaptive capabilities in LLMs [19]. Empirical studies show that incorporating empathetic behaviors can alleviate negative affective states [20,21] while enhancing the perceived competence and trustworthiness of conversational systems [15,22]. To enable such adaptation, structured response generation methods, including explicit planning of socioemotional strategies prior to text generation, have been proposed to improve both coherence and social appropriateness [23]. However, these improvements introduce new trade-offs. More empathetic responses are often longer and linguistically more complex, which may reduce accessibility for users with lower literacy levels [16,24]. This limitation is exacerbated by findings that standard LLMs exhibit limited sensitivity to user-specific education levels, often producing responses within a relatively narrow and fixed readability range, even when explicitly instructed to simplify [25]. Beyond generation, LLMs have also been shown to struggle to recognize the readability level of text, including their own outputs. A recent study found that LLM accuracy in classifying the reading level of generated responses declined substantially at higher complexity levels [26]. A comparable pattern has been observed in emotional recognition. While LLMs can approach expert-level reliability when judging clearly defined empathic content, their performance declines for more subtle, complex, or ambiguous emotional expression [27]. Oram et al [28] also analyzed LLMs’ consistency in evaluating their own emotionally driven responses and found reduced reliability specifically for moderately emotional text. This indicates that current state-of-the-art LLMs remain particularly unreliable at recognizing literacy and emotional cues in ambiguous cases. Recent approaches have therefore explored methods for controlling linguistic complexity, including instruction-tuning frameworks that enable more precise adjustment of readability and comprehension demands [29]. To prevent these challenges, conversational agents are commonly instructed to respond in a specific manner [30].

### Contribution and Plan of the Study

Despite these advances, there remains a lack of systematic evaluation of whether current LLMs can reliably adapt communication across emotional and literacy dimensions in patient-facing contexts and how such adaptations impact the usability of health interactions [19]. In particular, it is unclear whether these systems can simultaneously maintain contextual coherence, appropriate affective framing, and accessible information delivery when required to tailor responses to diverse user profiles. To date, this combined challenge has not been systematically investigated in the literature, making it difficult to assess the capabilities of current LLMs in this setting. This study addresses this gap by investigating the feasibility of using LLMs as adaptive conversational agents for health support, focusing on emotional sensitivity and literacy level simultaneously. To the best of our knowledge, this is the first study to examine this combination of adaptation dimensions in a single evaluation framework.

To isolate the effect of emotional and literacy adaptation from other sources of variability, we used a fully crossed, single-scenario experimental design. In this design, a single patient profile and a single question were held constant to provide consistent clinical context for the LLM’s responses and ensure that observed differences were attributable to the emotional and literacy adaptation rather than to variation in input content. Our objective was to determine whether current LLMs (Claude Opus 4.5, Gemini 3 Pro, Gemma 3 27B, and GPT 5.2) can maintain effective communication and internal coherence when prompted to adjust their affective tone and explanatory depth. For each model, we also analyze the trade-offs among model capacity, deployment configuration (cloud vs local), and the usability of the generated medical support.

To guide this investigation, we concentrated on the following research questions (RQs).

- RQ1: are LLMs capable of producing tailored responses across user profiles with varying emotional sensitivity and literacy levels? This question evaluated the effectiveness of controlled adaptation. LLM-generated responses were assessed through qualitative peer review to determine whether adaptations were perceptible, appropriate, and contextually meaningful. This is particularly important in patient-facing interactions, where well-adapted communication is more likely to be understandable, supportive, and actionable.
- RQ2: are LLMs consistent in evaluating both the emotional sensitivity and literacy level of input text and generating responses aligned with the same emotional and literacy categories? This question examined internal model coherence. Specifically, the same model re-evaluated its generated responses to determine whether it correctly identified the intended emotional and literacy levels. Consistency is critical because mismatches between generation and interpretation may indicate that a model has misinterpreted patients’ needs.
- RQ3: how do prompt verbosity and model architecture influence the quantitative properties (eg, length and verbosity) of the responses? This question explores a practical human factors trade-off between instruction depth and cognitive load placed on users during guidance. Even when responses are accurate and well-intentioned, excessive length or variability may reduce their usability.

## Methods

### Overview

The proposed methodology follows a multistage pipeline designed to evaluate the ability of LLMs to adapt communication and guidance to users’ emotional states and literacy levels. An overview of the methodology is illustrated in Figure 1.

**Figure 1:**
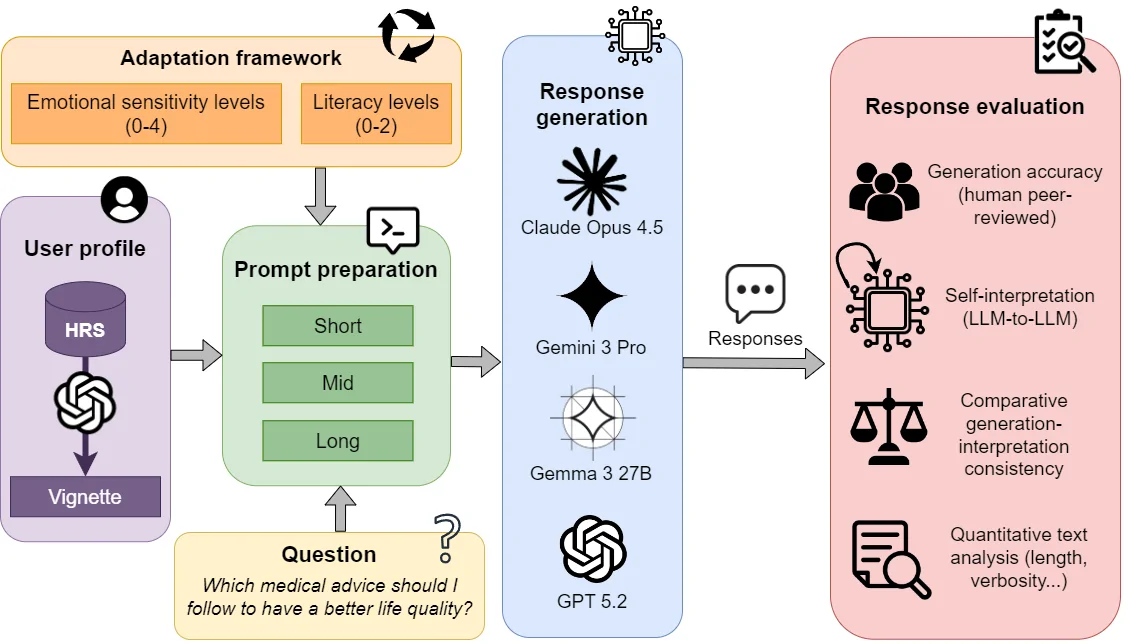
Details of the methodological phases of our approach. HRS: Health and Retirement Study; LLM; large language model.

First, we created a benchmark scenario based on realistic patient data. A representative user profile is generated from structured medical variables and summarized into a natural language description. Second, we defined controlled adaptation conditions across 2 dimensions (ie*,* emotional sensitivity and literacy level) using predefined scales, allowing granular classification and evaluation. Next, given a set of LLMs, we generated responses under different prompt configurations designed to elicit adaptive behavior. Subsequently, we evaluated the generated responses in terms of their alignment with the intended emotional and literacy characteristics and each model’s ability to interpret its own generated text. Finally, we studied the structural properties of the generated answers (eg*,* length and verbosity), providing insights into each model’s behavior.

To support reproducibility, all prompts, generated data, and evaluation materials are publicly available in a Zenodo (CERN [European Organization for Nuclear Research)] repository [31].

### User Profile Creation

To approximate a realistic patient context, we constructed a synthetic user profile based on variables commonly available in electronic health records in health care settings. Such profiles typically combine information on clinical history, cognitive status, and physiological measurements, which together were relevant for patient-centered communication and decision-making.

For this study, we derived this profile from the Health and Retirement Study (HRS), a large-scale longitudinal study of older adults in the United States [32]. Specifically, we used structured data from the HRS Core 2022 dataset [33], focusing on three relevant sections: (1) Section C, physical health, which includes diseases, diagnoses, medication use, lifestyle habits (eg*,* smoking, alcohol consumption, and sedentary behavior), and psychological status, (2) Section D, cognition, which captures measures of memory, orientation, and attention, and (3) Section I, physical measures, which provides objective indicators of physiological function, metabolic status, strength, and mobility.

From these data, we selected one participant with minimal missingness across the variables of interest. This choice helped maximize internal consistency across experimental conditions and ensure that the resulting profile is as complete as possible. The structured variables were then converted into a natural-language patient vignette using GPT 5.2, which was validated by the 3 authors (FC, AM-B, and EB). The vignette was provided as a standardized input in all experimental conditions, allowing the conversational agent to condition its responses on the same underlying health context while varying the instructed emotional and literacy levels. The vignette served a contextual rather than experimental function in the study design. It provided a realistic clinical context for the LLM’s responses rather than functioning as an independently manipulated variable.

### Question Design

The input question was designed to reflect the types of questions commonly posed by older adults, for whom factors such as social isolation and loneliness increase vulnerability to depressive symptoms and reduced well-being. In these settings, users may seek guidance not only on clinical aspects of care but also on broader concerns related to symptom management, quality of life, and self-care. Prior research on patient experience suggests a tendency to express concerns in an open-ended manner and seek holistic guidance that combines explanation, reassurance, self-management goals, and actionable advice, rather than formulating narrowly defined or binary questions [34-36]. These questions frequently involve issues related to meaning, self-understanding, and the need for recommendations that extend beyond purely clinical instructions. In addition to reflecting this tendency, using an open-ended question was methodologically necessary for this study. Narrow or highly specific questions could constrain the response space, limiting how much emotional tone and explanatory complexity could vary across conditions. In contrast, open-ended questions have been reported to elicit longer, more affectively rich, and more informative conversations than closed-ended ones [37,38].

We therefore used the following open-ended question to prompt the LLM-based conversational agent: “Which medical advice should I follow to improve my quality of life?” The question was intentionally formulated to be disease-agnostic, avoiding dependence on condition-specific medical knowledge and allowing the evaluation to focus on communication adaptation rather than clinical correctness. Using the same question across all experimental conditions enabled a controlled comparison of responses, with observed differences intended to reflect adaptation to the specified emotional and literacy levels rather than variations in the input question.

### Emotional and Literacy Adaptation Framework

To systematically evaluate adaptive communication, we defined two orthogonal dimensions: (1) emotional sensitivity and (2) literacy level. By considering these dimensions together across all experimental conditions, we evaluated the quality of the responses according to the model’s abilities to balance empathy, clarity, and appropriateness.

To the best of our knowledge, no existing instrument had been specifically designed to support discrete, prompt-based manipulation and evaluation of LLM-generated text across defined levels of emotional sensitivity and literacy. Existing related scales are often based on Likert-type ratings (eg*,* [27]), which tend to rely on subjective, loosely anchored descriptors that may be interpreted inconsistently by both human raters and LLMs. Consequently, the absence of an instrument offering well-defined and operationally anchored levels motivated the development of graded scales for both dimensions. The scales were iteratively developed by one author (AM-B), who sought to ensure that each level was conceptually distinct along a coherent gradient of emotional intensity or literacy complexity. The other 2 authors (FC and EB), who applied the scales during the evaluation process, independently reviewed and validated them to ensure that the level definitions were clear, unambiguous, and sufficiently differentiated.

The emotional adaptation scale consists of 5 levels (0-4), ranging from harsh and factual communication to highly supportive responses characterized by empathy, reassurance, and emotional validation. The literacy adaptation scale comprises 3 levels (0-2), ranging from simplified, step-by-step explanations with minimal jargon to technical and nuanced descriptions. The 2 scales differ in granularity because the underlying dimensions do not lend themselves equally to fine-grained subdivision: emotional sensitivity spans a wide behavioral and affective gradient, which supports a richer set of clinically meaningful distinctions, whereas literacy is more naturally bounded by a smaller number of broadly distinguishable comprehension levels, beyond which further subdivision risks introducing arbitrary or poorly distinguishable categories rather than added explanatory value [26]. The operational criteria for prompting and evaluation are described for both dimensions in Textbox 1 and Textbox 2, respectively. This framework allowed us to assess whether models could adapt along each dimension individually and, crucially, whether they could preserve coherence when both emotional and literacy requirements are imposed simultaneously within the same response.

**Definitions of the emotional adaptation levels: short variant.**

Emotional adaptation levels are as follows:

- Level 0: very rude or harsh tone; prefers blunt, concise, and highly factual responses; easily annoyed by emotional language.
- Level 1: slightly irritable; prefers direct language but tolerates some nuance; dislikes overly emotional or vague explanations.
- Level 2: neutral to moderately sensitive; prefers a calm, respectful tone; appreciates straightforward but kind language.
- Level 3: sensitive or easily discouraged; prefers a supportive and affirming tone; avoids confrontation or stress-inducing phrasing.
- Level 4: very emotionally vulnerable or anxious; needs a high level of empathy and emotional reassurance in all communication.

**Definitions of the literacy adaptation levels: short variant.**

Literacy adaptation levels are as follows:

- Level 0: very low literacy or cognitive capacity; requires simple, step-by-step explanations using basic vocabulary and repetition.
- Level 1: moderate comprehension; capable of processing moderately complex explanations with some simplification.
- Level 2: high cognitive ability; highly literate and comfortable with abstract and technical concepts.

### Prompt Variants

To investigate how instruction design influences adaptive behavior, we used 3 prompt variants with increasing verbosity: short, medium, and long. All variants instructed the model to tailor its response according to the specified emotional and literacy levels. However, they differ in the granularity and explicitness of these instructions. The short variant provided minimal guidance and served as a baseline for assessing whether LLMs can perform adaptation with limited instruction. The medium and long variants provided progressively more detailed descriptions of the adaptation criteria. All prompts used for response generation are provided in Multimedia Appendix 1. This design enabled us to evaluate whether increased prompt specificity improved alignment with the intended communication style or whether concise prompts are sufficient for effective adaptation. It also allowed us to analyze trade-offs among prompt length, response quality, and computational efficiency, which are relevant for real-world deployment.

In addition, we used a separate set of self-evaluation prompts to assess the generated outputs and examine whether LLMs could consistently judge their own responses under matched prompt conditions. All prompts used for self-evaluation are provided in Multimedia Appendix 2.

### Experimental Setup for Response Generation

Each LLM was systematically evaluated across all combinations of emotional sensitivity levels (5 levels), literacy levels (3 levels), and prompt variants (3 lengths), resulting in 45 distinct response conditions (5×3×3). To minimize potential bias, each generation prompt (see Multimedia Appendix 1) was issued independently.

To facilitate comparison and establish a performance baseline, we deliberately restrict our evaluation to 4 specific LLMs (Table 1): 3 state-of-the-art commercial cloud-based models (Claude Opus 4.5, Gemini 3 Pro, and GPT 5.2) and one medium-scale model that could be deployed locally (Gemma 3 27B). This selection was intentional. Adapting to simultaneous emotional and literacy constraints represents a complex zero-shot reasoning task. Our objective was to determine the performance of current generative AI before extending the evaluation to smaller, more constrained open-source models, because we considered it important to establish whether the selected advanced models could reliably manage this multidimensional adaptation. Furthermore, Gemma 3 27B was included as an intermediate comparison, without using models with fewer parameters that historically struggle with complex instruction following [39,40].

**Table 1:** Large language models used in the experimental setup.

Name	Specific model name	Developer	Setup
Claude Opus 4.5	claude-opus-4-5-20251101	Anthropic	Cloud
Gemini 3 Pro	gemini-3-pro-preview	Google	Cloud
Gemma 3 27B	gemma3:27b	Google	Local
GPT 5.2	gpt-5.2-chat-latest	OpenAI	Cloud

All experiments were executed in a Google Colab environment, interfacing with the local model via an Ollama server (local API endpoint) and with the commercial models through their respective APIs. Overall, the experimental setup yielded 180 responses in total across all models (45×4). All models produced valid outputs followed the required response structure, and no execution errors were observed.

### Evaluation Protocol for Response Evaluation

The evaluation protocol was designed to systematically assess the ability of LLMs to generate and interpret medically relevant conversational responses under controlled emotional and literacy adaptation conditions. In total, 180 responses were evaluated using four complementary approaches: (1) generation-based evaluation, (2) self-interpretation evaluation, (3) comparative generation-interpretation consistency analysis, and (4) quantitative text analysis.

First, generation performance was assessed by measuring the alignment of responses with the intended emotional and literacy levels. This alignment was established through independent human ratings followed by consensus resolution. Two authors (FC and EB) independently rated each of the 180 responses for alignment with the target levels. Disagreements (23/180, 12.8%) were resolved through consensus to establish the final human-reviewed ground truth values. Most disagreements concerned the emotional scale and involved intermediate values (eg, 1 vs 2). No disagreements occurred for literacy ratings or extreme emotional levels. To ensure methodological consistency, the same definitions used during the generation phase were applied during rating. Alignment was quantified using the mean absolute error (MAE) between the target levels and the human-reviewed reference values. This evaluation captured how accurately each model produced responses that match the intended adaptation specifications.

Second, interpretation performance was evaluated in a self-assessment setting, in which each model was prompted to classify the emotional and literacy levels of its own previously generated responses. To ensure consistency, the same definitions and prompting formulations used during generation were reused during self-evaluation (see Multimedia Appendix 2). Thus, each model evaluated only its own set of 45 generated responses across all prompt variants, assigning both emotional and literacy labels to its outputs. These outputs were then compared with the human-reviewed reference values to quantify the error. Direct cross-model evaluation was not included in this setting, as LLM-generated responses do not constitute an independent reference standard for assessing other models’ judgments, making such comparisons difficult to interpret. Consequently, the results of this self-evaluation should not be interpreted as a measure of objective correctness in a human-validated sense but rather as an indication of each model’s internal consistency and ability to interpret its own outputs within the defined adaptation framework. This design allowed us to assess how consistently each model interpreted its own responses under the same adaptation framework.

Third, a generation-interpretation consistency analysis was conducted to examine the relationship between a model’s ability to produce responses at a given adaptation level (generation) and its ability to subsequently identify those levels (interpretation). Thus, we compared the generated and interpreted emotional and literacy levels with the corresponding levels validated by the authors to calculate task-specific accuracy. This experiment provided insight into the internal coherence of adaptation behavior across generation and interpretation tasks.

Fourth, quantitative text analysis was performed to examine the structural properties of the generated responses, with a particular focus on response length and verbosity. This analysis examined how emotional and literacy adaptation levels, as well as prompt verbosity, influenced the textual characteristics of generated medical responses.

### Ethical Considerations

This research involved secondary analysis of deidentified HRS data that were publicly available upon registration. The dataset did not contain direct participant identifiers, and the analyses were conducted on deidentified data without any attempt to identify individual participants. In accordance with the applicable HRS terms and conditions of use, this study did not require ethical review.

## Results

### Overview

This section presents the empirical results of the evaluation protocol according to the 4 complementary analyses in the experimental setup described above.

### Generation-Based Evaluation

The results show varying degrees of alignment between model responses and the human-reviewed ground-truth, as summarized in Table 2. Overall, literacy adaptation was more consistent than emotional adaptation across all evaluated models. Literacy errors were generally low and reached zero in several configurations, suggesting that models could adjust explanatory depth, vocabulary, and informational density under the tested conditions. In contrast, emotional adaptation showed greater variability, suggesting that affective tone was more difficult for the models to control consistently.

**Table 2:** Accuracy of the generation-based evaluation.

Model and prompt variant		Emotion MAE^a^, mean (SD)	Literacy MAE, mean (SD)
Claude Opus 4.5			
	Long	0.20 (0.41)	0.00 (0.00)
	Middle	0.13 (0.35)	0.00 (0.00)
	Short	0.20 (0.41)	0.07 (0.26)
Gemini 3 Pro			
	Long	0.13 (0.35)	0.07 (0.26)
	Middle	0.20 (0.41)	0.00 (0.00)
	Short	0.13 (0.35)	0.00 (0.00)
Gemma 3 27B			
	Long	0.13 (0.35)	0.07 (0.26)
	Middle	0.07 (0.26)	0.07 (0.26)
	Short	0.47 (0.52)	0.07 (0.26)
GPT 5.2			
	Long	0.27 (0.46)	0.00 (0.00)
	Middle	0.20 (0.41)	0.00 (0.00)
	Short	0.27 (0.46)	0.07 (0.26)

^a^MAE: mean absolute error.

Among the evaluated models, Gemma 3 had the lowest emotional MAE (mean 0.07, SD 0.26, under the middle-length prompt) but also exhibits the highest variability, particularly under the short prompt condition, where error increased to mean 0.47, SD 0.52. This suggested that local models were capable of precise affective adaptation under detailed instructions but were less robust when prompt guidance was limited. In comparison, Claude Opus 4.5, Gemini 3 Pro, and GPT 5.2 showed more stable performance overall. These models had consistently low literacy error across all prompt variants (averages around 0.00-0.07), while their emotional error remains relatively contained (averages around 0.13-0.27). In particular, Claude Opus 4.5 and GPT 5.2 had its lowest emotional MAE under the short prompt. By contrast, Gemini 3 Pro had its lowest emotional MAE under the short prompt. These results indicate that prompt length did not improve generation performance uniformly across models. Instead, each model appeared to perform best under a different balance of conciseness and specificity, highlighting the importance of prompt design in adaptive communication tasks.

These results suggest that, under the tested conditions, current LLMs produced literacy-adapted responses with relatively low MAE according to human ratings, whereas emotional adaptation was more variable and model-dependent. This distinction is especially relevant for health care communication applications, where responses may be informationally appropriate yet still affectively misaligned.

### Self-Interpretation Evaluation

Self-interpretation results showed differences across models in their ability to identify the reference emotional and literacy levels of their own responses, as summarized in Table 3. Overall, models showed different levels of performance in emotional and literacy interpretation.

**Table 3:** Accuracy of the self-interpretation evaluation according to the human-reviewed ground truth level.

Model and prompt variant			Emotion MAE^a^, mean (SD)		Literacy MAE, mean (SD)
Claude Opus 4.5					
	Long	0.80 (0.86)		0.07 (0.26)	
	Middle	1.00 (0.76)		0.07 (0.26)	
	Short	1.13 (1.06)		0.13 (0.35)	
Gemini 3 Pro					
	Long	0.60 (0.74)		0.13 (0.35)	
	Middle	0.60 (0.74)		0.00 (0.00)	
	Short	0.80 (0.77)		0.33 (0.62)	
Gemma 3 27B					
	Long	0.73 (0.80)		0.67 (0.49)	
	Middle	0.93 (0.80)		0.60 (0.51)	
	Short	0.73 (0.59)		0.67 (0.49)	
GPT 5.2					
	Long	0.53 (0.64)		0.53 (0.64)	
	Middle	0.80 (0.77)		0.60 (0.63)	
	Short	0.67 (0.62)		0.87 (0.83)	

^a^MAE: mean absolute error.

For emotional interpretation, Gemini 3 Pro and GPT 5.2 had achieved the lowest errors, indicating stronger capability in recognizing affective tone. Gemini 3 Pro emerged as the most balanced model across prompt variants (averages around 0.60-0.80), maintaining relatively stable performance under middle and long prompts, whereas GPT 5.2 showed its best performance under the long prompt condition. In contrast, Claude Opus 4.5 exhibited the worst emotional interpretation, particularly for short and middle prompts (averages around 1.00-1.13), while Gemma 3 27B maintained moderate but less consistent performance across all configurations. Literacy interpretation errors revealed a reversed pattern. Claude Opus 4.5 achieved consistently low literacy error across all prompt variants (averages around 0.07-0.13), indicating strong reliability in recognizing explanatory depth and vocabulary complexity. Gemini 3 Pro also performed well, achieving perfect literacy accuracy under the middle prompt. By contrast, GPT 5.2 showed substantially higher and more variable literacy errors (averages around 0.53-0.87), and Gemma 3 27B also presented comparatively elevated errors. These results suggested that both models face greater difficulty in self-recognizing the literacy levels.

Model-specific asymmetries were evident; Claude Opus 4.5 demonstrated stronger performance in literacy interpretation but weaker accuracy in emotion recognition, whereas GPT 5.2 followed the opposite tendency. Gemma 3 27B faced particular challenges in literacy self-assessment despite comparatively stable emotional interpretation. Finally, the effect of prompt length varied across models or dimensions, and no single prompting strategy emerged as universally optimal for self-interpretation tasks.

### Level-Wise Comparative Generation-Interpretation Consistency

Results showed a clear asymmetry between the models’ capabilities in generating responses at a target adaptation level and in interpreting them according to the ground truth level, as summarized in Tables 4 and 5, respectively.

**Table 4:** Average MAE^a^ by target level (generation).

Dimension	Level 0 MAE, mean (SD)	Level 1 MAE, mean (SD)	Level 2 MAE, mean (SD)	Level 3 MAE, mean (SD)	Level 4 MAE, mean (SD)
Emotional sensitivity	0.056 (0.232)	0.250 (0.439)	0.333 (0.478)	0.139 (0.350)	0.222 (0.421)
Literacy	0.017 (0.129)	0.000 (0.000)	0.083 (0.278)	—^b^	—

^a^MAE**:** mean absolute error.

^b^Not applicable.

**Table 5:** Average MAE^a^ by level according to the human-reviewed ground truth (interpretation).

Dimension	Level 0 MAE, mean (SD)	Level 1 MAE, mean (SD)	Level 2 MAE, mean (SD)	Level 3 MAE, mean (SD)	Level 4 MAE, mean (SD)
Emotional sensitivity	1.634 (0.829)	0.921 (0.358)	0.516 (0.569)	0.238 (0.431)	0.429 (0.503)
Literacy	0.559 (0.701)	0.424 (0.498)	0.164 (0.373)	—^b^	—

^a^MAE**:** mean absolute error.

^b^Not applicable.

In the response generation task, higher variability was observed when models targeted intermediate emotional levels (ie*,* Level 2), where the error rate peaked at 0.333. This suggests that an intermediate affective tone may be less clearly defined and more susceptible to subjective variation, potentially reflecting differences in training data and in the internal representation of tone. In addition, adjacent emotional levels may be difficult to distinguish during generation, indicating limited granularity in emotional synthesis. For literacy, although overall error rates remained low (below 0.083), some models showed difficulty in generating highly technical responses, suggesting limitations in reaching higher levels of literacy complexity and technical depth.

In contrast, the interpretation task showed an inverse pattern. MAEs were highest for Level 0 emotional sensitivity (1.634) and Level 0 literacy (0.559), indicating a systematic bias toward interpreting such responses as the middle of the scale rather than their reference levels. These findings suggest that models had greater difficulty identifying low emotional-sensitivity and literacy levels than generating responses at those levels.

This divergence between generation and interpretation may reflect a limitation of current LLMs. Inconsistent alignment between these 2 processes may reduce the effectiveness of adaptive conversational agents. For example, misclassification of low-literacy or low-emotional-sensitivity levels may lead a system to default to more neutral response strategies, thereby reducing accessibility and the perceived relevance and supportiveness of the interaction.

### Quantitative Text Analysis

Results revealed consistent patterns in response length across models and prompt variants. As shown in Figure 2, response length generally increased at higher emotional sensitivity levels. This trend suggests that models generate more elaborate and expansive responses when higher emotional sensitivity is required, likely reflecting increased use of supportive and reassuring language. A similar pattern was observed across literacy levels, with responses becoming longer at higher literacy levels, suggesting that models allocated more space to explanations with greater technical depth and informational detail for higher-literacy users.

**Figure 2:**
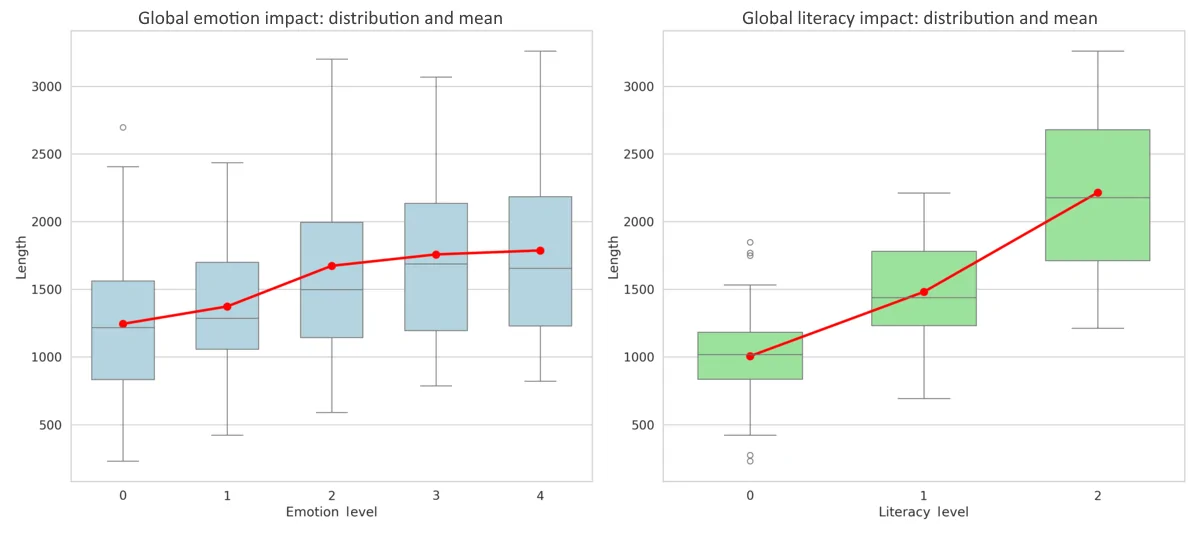
Distribution of response length across emotional sensitivity and literacy levels, models, and prompt variants.

More specifically, Figures 3 and 4 showed that prompt design had additional but comparatively moderate effects on response length. The long prompt variant generally produced the longest responses across both emotional and literacy levels, particularly at higher values. However, the overall variation in mean response length across prompt variants remains limited, suggesting that the requested emotional and literacy levels may have been the primary drivers of response length, while prompt length may have acted as a secondary modulator.

**Figure 3:**
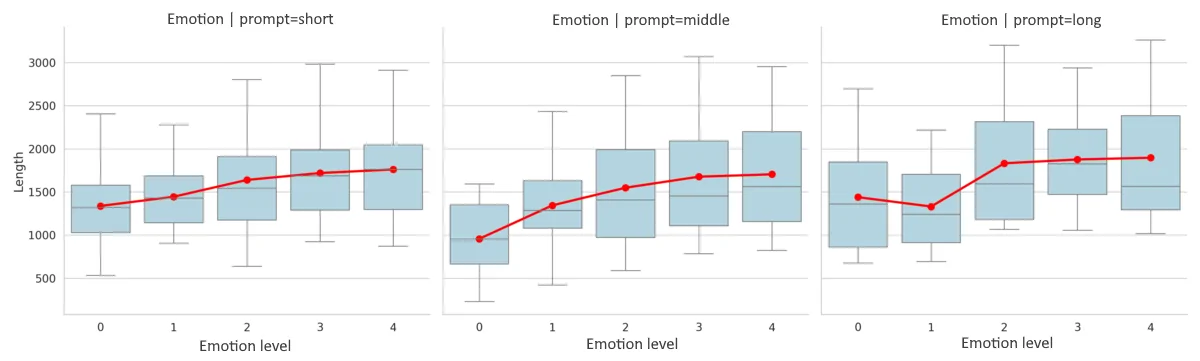
Distribution of response length across emotional sensitivity levels and models by prompt variant.

**Figure 4:**
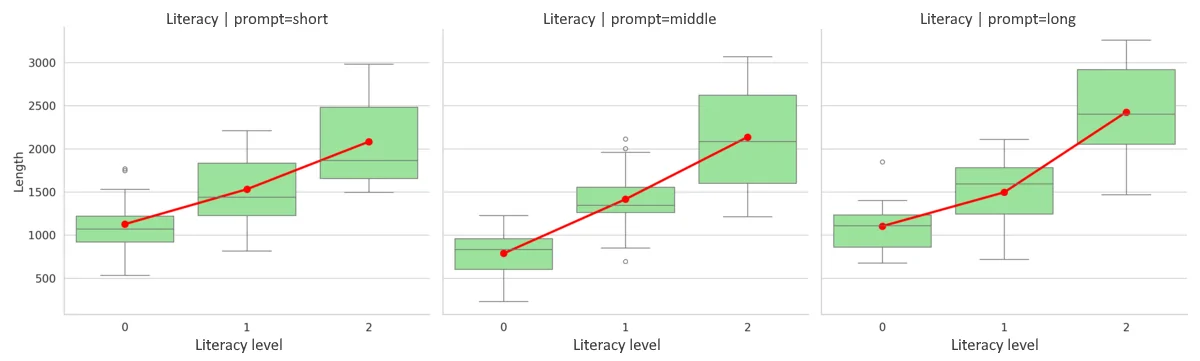
Distribution of response length across literacy levels and models by prompt variant.

Figure 5 showed clear model-specific differences in generative behavior. While short and middle-length prompts produced broadly comparable response lengths across models, the long prompt increased both the mean and variability of responses. Notably, Gemini 3 Pro showed substantial variability in response length across all prompt variants, indicating a less constrained generation pattern. Claude Opus 4.5 exhibited a more pronounced sensitivity to prompt length, with a marked increase in verbosity under the long prompt compared to shorter variants. In contrast, GPT 5.2 consistently generated the most verbose responses overall, with particularly high dispersion under the middle-length prompt variant.

**Figure 5:**
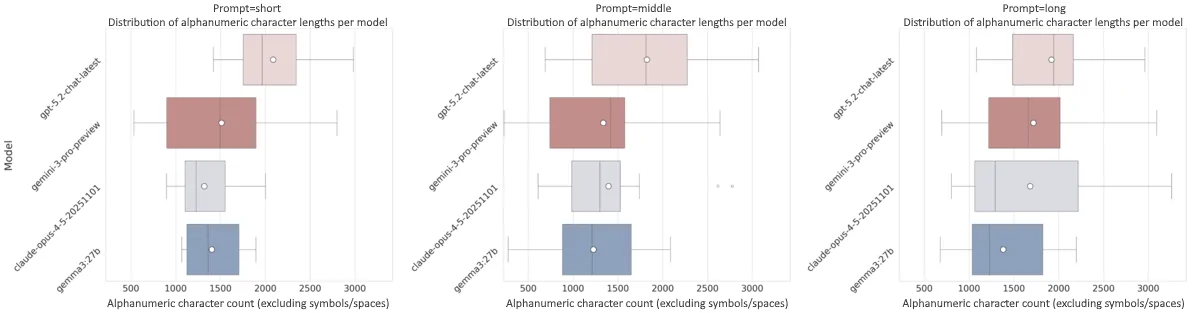
Distribution of alphanumeric character counts by prompt variant and model.

Together, these findings indicate that response length in conversational agents is associated with (1) target emotional and literacy levels, (2) prompt verbosity, and (3) model-specific generation tendencies. This relationship is relevant for digital health applications, where model choice may influence the nature and structure of responses.

## Discussion

This study provides a proof-of-concept characterization, within a controlled single-scenario design, of the interactional capabilities of contemporary LLMs by investigating the extent to which they can adapt communication to emotional sensitivity and literacy levels in patient-facing settings. Overall, the findings indicate that such adaptation is feasible but varies across dimensions, models, and prompting strategies. In particular, the results highlight systematic differences between literacy adaptation and emotion adaptation, as well as important limitations in how models generate and interpret user-aligned responses.

With respect to RQ1, the human-reviewed ground-truth evaluation showed that LLMs consistently achieve high accuracy in literacy adaptation, often reaching zero (or near-zero) errors in higher-capacity models like Claude Opus 4.5. This indicates that these models are capable of reducing the literacy load on patients by distilling technical medical advice into accessible, step-by-step instructions. From a safety perspective, this is a vital finding, as reducing the “barrier to information” is a primary goal of inclusive health technology design [41]. In contrast, emotional adaptation was more variable and strongly model-dependent. Although models generated empathetic language, their ability to maintain consistent affective alignment across conditions appeared less reliable. For a highly sensitive user, a model that prioritizes data-driven responses over de-escalation may pose a communication-safety risk. This pattern was particularly evident at intermediate emotional levels, where variability was higher, suggesting that intermediate emotional tones may be harder to define and reproduce consistently. Our findings align with prior evidence indicating that, while LLMs can generate supportive language, their responses are overwhelmingly neutral or inconsistently compassionate compared with those of human physicians [24,42].

Another key finding is the asymmetry between generation and interpretation, which suggests a gap in consistency between these tasks (RQ2). Models that perform well in generating simplified or emotionally adapted responses do not necessarily recognize those characteristics equally well in their own responses. In particular, although models generated responses with low complexity and low literacy-level MAEs, they had greater difficulty identifying low-literacy levels in their own responses. Thus, an LLM may generate simple language but may not reliably identify when a user requires it. Lower levels of literacy and emotional sensitivity were frequently associated with higher interpretation errors, suggesting a possible tendency toward middle-range classifications. This misalignment may indicate that current LLMs do not consistently represent human-centered communication dimensions across generation and interpretation, which could lead to misinterpretation in fully automated medical dialogues. This raises an equity concern: users who may benefit most from adapted communication, such as those with lower literacy, may be at greater risk of being misclassified by these systems.

The findings also revealed differences across models. Larger cloud-based models, particularly Gemini 3 Pro, showed lower interpretation MAEs across the evaluated prompt variants, but these results do not establish that the models have more stable internal representations of the adaptation criteria. Although Gemma 3 27B is substantially smaller than the other models evaluated, its performance on certain tasks was comparable to that of its larger counterparts, particularly for emotional self-interpretation. However, performance varied by the prompt variant, with significant differences in tasks such as the generation of responses with adequate emotional sensitivity. This implies that for privacy-sensitive applications, where local models are preferred, developers must implement more rigorous prompt-based safeguards to ensure the models do not drift into inappropriate tones. This trade-off between deployment autonomy (privacy) and adaptive precision (safety) is a critical design decision for future geriatric or mental health assistive tools [43]. Additionally, accountability concerns arise when a conversational agent behaves inconsistently across generation and interpretation. In such cases, it may be harder to determine whether responsibility for resulting miscommunication lies with the developer, deployer, or clinician.

Regarding RQ3, the comparison between the locally deployed model and the cloud-based models, together with the quantitative text analysis, suggests a potential trade-off between adaptation richness and deployment constraints. The evaluated cloud-based models generally produced more elaborate responses in some conditions, although computational requirements and data locality were not directly evaluated in this study. Conversely, smaller models offer advantages in efficiency and privacy but require proper prompting strategies to maximize their effectiveness. Indeed, as even the most advanced LLMs struggle with emotional generation and interpretation, evaluating such small-scale LLMs, including mixture-of-experts models [44], under identical zero-shot conditions, is likely to yield less adaptable responses. These differences suggest that deployment decisions in health care settings should balance not only performance and adaptation but also privacy, cost, and controllability aspects.

The analysis of response length further complements these findings. Response verbosity increased with both emotional sensitivity and literacy level, indicating that models naturally expand their responses when more supportive or more detailed communication was required. However, verbosity length also varied across models and prompt variants, with some models (eg*,* Gemini 3 Pro) producing substantially more variable responses than their counterparts. While longer responses may benefit highly literate users, they risk overwhelming users with lower literacy. Our findings suggest that the middle-length prompt variant often serves as the optimal design intervention, providing enough contextual guidance for the model [45]. This reinforces the need to consider not only correctness and adaptation but also conciseness and usability when designing conversational agents.

This study had several limitations. First, the evaluation deliberately used a controlled single-scenario design (ie*,* one user profile, one question, and a fixed set of predefined prompts and adaptation levels) to isolate the effects of emotional and literacy adaptation from other sources of variability. This design increased control over the experimental conditions but limited generalizability. The results may not generalize to the full diversity of potential real-world questions, profiles, and conversational contexts. Second, the lack of existing scales specifically designed to operationalize discrete levels of emotional sensitivity and literacy for LLMs led us to develop our own scales, as Likert-like scales were discarded for being too ambiguous. In their current form, these scales are not intended to serve as universally applicable instruments for assessing LLM communication; rather, they provide a way to systematically assess how LLMs adapt their responses across clearly delineated levels. Finer-grained or more numerous levels might offer a richer characterization of adaptive behavior but could make the distinctions harder to detect reliably, particularly for smaller models. Third, human evaluation, while structured, remains inherently interpretive and may introduce subjective bias. For this reason, consensus procedures were adopted in cases of disagreement. Fourth, the analysis focused on controlled one-shot generations rather than longer multiturn dialogues, so it could not fully capture how adaptation evolves over time. Finally, the assessments made are not fully replicable due to the inherent variability of the models in generating responses, which is a general limitation of LLMs. To minimize the impact of the latter, our data [31] provides the full set of outcomes and not only the set of generated responses.

In conclusion, within our proof-of-concept scenario, contemporary LLMs demonstrated the capacity to adapt communication to patients’ emotional and literacy profiles, although this capability was uneven and highly context-dependent. These findings should be interpreted as evidence of adaptive capacity under controlled conditions rather than as a generalizable estimate of performance across the full range of real-world variants. While literacy adaptation emerged as a robust strength, emotional alignment and interpretation were less stable, and the observed gap between generation and interpretation highlights important limitations in current model behavior. These findings suggest that the design of conversational agents may benefit from leveraging multiple models, each specialized in tasks in which they perform most reliably. More importantly, such systems should be positioned as assistive communication tools rather than autonomous decision-makers, particularly in sensitive domains such as mental health. Their effective use would require human oversight, careful system design, and explicit safeguards. Moreover, ethical and privacy considerations remain central. The handling of sensitive health-related information, the risk of overreliance on automated outputs, and the need to inform patients clearly when they are interacting with an AI system rather than a human clinician all necessitate cautious, transparent, and accountable deployment.

Future research should extend this framework in several directions. First, evaluating smaller and more efficient models will be important to better understand trade-offs among performance, cost, and deployment practicality. Second, further work is needed to investigate mechanisms for controlling response length and improving conversational efficiency without compromising emotional and literacy adaptation. Third, expanding the evaluation to not only more questions, more profiles, and other scales, but also to multilingual settings, will help determine whether these findings generalize across linguistic and cultural contexts. Fourth, developing standardized scales for assessing LLMs is needed to foster comparison among other studies. Finally, studying multiturn interactions will be essential to assess how adaptive communication evolves over time in realistic health care scenarios.

## Data Availability

To support reproducibility, all prompts, generated data, and evaluation materials are publicly available in a Zenodo repository [31]. However, no participant-level Health and Retirement Study data are included in the repository, in accordance with Health and Retirement Study terms and conditions of use.

## Appendix

Multimedia Appendix 1 Response generation prompts.

Multimedia Appendix 2 Self-evaluation prompts.

## Abbreviations

CERN: European Organization for Nuclear Research
GenAI: generative AI
HRS: Health and Retirement Study
LLM: large language model
MAE: mean absolute error
RQ: research question
