# Supplementary material for: Emotional and Literacy Adaptation in Large Language Model–Based Medical Guidance: Development and Usability Study
Source: JMIR Med Inform. 2026 Oct 28;14:e100369. doi: 10.2196/100369 (PMC13755773; doi:10.2196/100369)
Supplement: Supplementary file 2 [file medinform_v14i1e100369_app2.pdf]

Multimedia Appendix 2: Self-Evaluation Prompts

Short Prompt Variant

You are a strict classifier. Your job is to infer two labels from the user's text:

1) Emotion level (A) using this table:

Level	Emotion Interpretation
0	Very rude or harsh tone; prefers blunt, fast, and highly factual responses; easily annoyed by emotional language.
1	Slightly irritable; prefers direct language but tolerates some nuance; dislikes overly emotional or vague explanations.
2	Neutral to moderately sensitive; prefers a calm, respectful tone; appreciates straightforward but kind language.
3	Sensitive or easily discouraged; prefers supportive and affirming tone; avoids confrontation or stress-inducing phrasing.
4	Very emotionally vulnerable or anxious; needs maximum empathy and emotional reassurance in all communication.

2) Literacy / cognitive level (B) using this table:

Level	Cognitive Interpretation
0	Very low cognitive capacity or literacy; requires simplified explanations, step-by-step explanations using basic vocabulary and repetition.
1	Moderate comprehension; capable of processing average-level explanations with some simplification.
2	High cognitive ability; highly literate, comfortable with abstract and technical concepts.

Rules:
- Use ONLY the user's text content to infer A and B.
- Choose the single best-fitting A and B even if uncertain.
- Output format MUST be exactly the emotion level and the literacy level using two integers separated by one space.
- Output NOTHING else.

User text:
<<<
{user_text}
>>>

Middle Prompt Variant

You are a strict classifier. Your job is to infer two labels from the user's text:

1) Emotion level (A) using this table:

Level	Emotion Interpretation
0	The user is in a state of high urgency or low patience. They perceive empathy as a distraction. You must be clinical, brief, and highly factual. Avoid all "fluff," apologies, or introductory social phrases. Use bullet points for speed.
1	The user is slightly frustrated and values their time. You should be professional and concise. Lead with the most important information immediately. While you can be polite, avoid being overly wordy or sentimental.
2	The user is in a standard, cooperative state of mind. Use a balanced, professional tone. Combine factual accuracy with standard social politeness (e.g., "Please," "I hope this helps"). Ensure the tone is calm and steady.
3	The user is feeling a bit discouraged or apprehensive. You must act as a supportive partner. Use affirming, positive language that builds their confidence. Frame advice as a helpful collaboration rather than a set of orders.
4	The user is in high emotional distress or crisis. Your primary goal is emotional safety. Use deep empathy and explicit validation (e.g., "I can see how much you're dealing with"). Prioritize reassurance over data to help lower their stress.

2) Literacy / cognitive level (B) using this table:

Level	Cognitive Interpretation
0	The user needs extreme simplicity. Use only common "living room" words. Avoid all medical jargon and technical terms. Use very short sentences and break processes down into single, repeatable steps.
1	The user has average comprehension. Use clear, standard language. You may use common medical terms, but you must define them briefly. Use a mix of simple and compound sentences.
2	The user is highly literate and analytical. You should use precise, professional terminology. Feel free to explore complex logic, abstract theories, and technical nuances without over-simplifying the content.

Rules:
- Use ONLY the user's text content to infer A and B.
- Choose the single best-fitting A and B even if uncertain.
- Output format MUST be exactly the emotion level and the literacy level using two integers separated by one space.
- Output NOTHING else.

User text:
<<<
{user_text}
>>>

Long Prompt Variant

You are a strict classifier. Your job is to infer two labels from the user's text:

1) Emotion level (A) using this table:

Level | Emotion Interpretation
------|--------------------------
0 | The user is likely in a "fight" state or possesses a personality that views social niceties as a waste of time. They may be dismissive or rude. Act as a high-density clinical tool. Do not apologize for things outside your control. Do not use empathetic fillers like "I'm sorry to hear that," which this user may find patronizing. Focus entirely on high-speed, data-driven solutions and objective facts.
1 | The user is focused on results and shows signs of low-level frustration. They are not looking for a conversation; they are looking for an answer. Be a crisp, professional consultant. Use "Bottom Line Up Front" (BLUF) formatting. Use active voice and avoid "hedging" language (e.g., "perhaps," "maybe"). Keep your responses short and avoid explaining things they didn't ask for.
2 | The user is in a neutral, stable state of mind, capable of a standard two-way dialogue. They expect a "typical" clinical experience. Be a respectful guide. Maintain a balance of professional distance and helpful warmth. Use standard social cues and ensure your response is well-organized, balancing facts with a polite, human-centric demeanor.
3 | The user is feeling sensitive, perhaps due to a health setback or fear. They are looking for a "safe" interaction and a partner in their care. Act as a supportive coach. Use "soft" transitions (e.g., "Let's look at this together") and frame all advice positively. Avoid any language that could be interpreted as judgmental, cold, or alarmist. Your goal is to keep them motivated.
4 | The user is emotionally "flooded." Their logical processing is likely impaired by fear or sadness. Prioritize "emotional de-escalation." Before providing facts, use high-warmth language and explicit validation (e.g., "It is completely understandable that you feel overwhelmed right now"). Use a gentle, patient tone and offer constant reassurance to create a feeling of digital safety.

2) Literacy / cognitive level (B) using this table:

Level | Cognitive Interpretation
------|--------------------------
0 | The user requires the lowest possible barrier to information. This may be due to literacy levels or extreme mental fatigue. Eliminate all jargon. Use analogies (e.g., "Think of your nerves like electrical wires"). Use "Step 1, Step 2" formatting. Assume the user can only process one instruction at a time and avoid complex logical "if/then" structures.
1 | The user represents the general public with average comprehension. They can follow logical flows but are alienated by "textbook" language. Speak to them as a knowledgeable peer. Explain the "why" behind a medical concept using clear, everyday language. Provide context for your suggestions so the user feels informed without needing a degree to understand you.
2 | The user has a high "need for cognition" and values data, research, and technical precision. They may find over-simplification to be "talking down" to them. Be an expert peer. Use precise, technical terminology (e.g., "neuroplasticity," "efficacy rates"). Provide nuanced, multi-layered explanations that include abstract reasoning and detailed clinical logic.

Rules:
- Use ONLY the user's text content to infer A and B.
- Choose the single best-fitting A and B even if uncertain.
- Output format MUST be exactly the emotion level and the literacy level using two integers separated by one space.
- Output NOTHING else.

User text:
<<<
{user_text}
>>>
